# The Vicious Worm education tool improves the knowledge of community health workers on *Taenia solium* cysticercosis in Rwanda

**DOI:** 10.1371/journal.pntd.0012140

**Published:** 2024-04-17

**Authors:** Esther Uwibambe, Anselme Shyaka, Egide Niyotwagira, Justine Mutoniwase, Eric M. Fèvre, Rupert J. Quinnell, Chiara Trevisan

**Affiliations:** 1 Center for One Health, University of Global Health Equity, Butaro, Rwanda; 2 School of Medicine, University of Global Health Equity, Butaro, Rwanda; 3 Institute of Infection, Veterinary and Ecological Sciences, University of Liverpool, United Kingdom; 4 International Livestock Research Institute, Nairobi, Kenya; 5 Faculty of Biological Sciences, School of Biology, University of Leeds, Leeds, United Kingdom; 6 Department of Translational Physiology, Infectiology and Public Health, Faculty of Veterinary Medicine, Ghent University, Belgium; 7 Department of Public Health, Institute of Tropical Medicine, Antwerp, Belgium; UDLA: Universidad de Las Americas, ECUADOR

## Abstract

The pork tapeworm *Taenia solium* causes human taeniasis and cysticercosis when ingested as viable cysts and eggs, respectively. Despite its high health burden in low-income countries, knowledge of the parasite in endemic areas such as Rwanda is often limited. Here, we assess whether The Vicious Worm education software can increase knowledge in endemic areas of Rwanda. A cross-sectional mixed-methods study was conducted to evaluate knowledge about *T*. *solium* among community health workers trained using the Vicious Worm education software. Knowledge was assessed before, immediately after, and four weeks after the training. The health workers perceptions of the software were analysed thematically. A total of 207 community health workers were recruited from Nyamagabe district in Southern Province, Rwanda. Participants were composed of males (33.5%) and females (66.5%), aged between 22 and 68 years, and most (71%) had only completed primary education. Knowledge of cysticercosis at baseline was low, particularly knowledge of human cysticercosis and neurocysticercosis. The overall knowledge score increased significantly after training and was maintained four weeks after the training. Overall, insufficient knowledge was associated with neurocysticercosis-related questions, which after the training, remained relatively lower compared to questions of other categories. Participants reported the software to be user-friendly and educational. Digital illiteracy and the lack of smartphones were among the critical challenges highlighted in responses. This study has shown gaps in knowledge regarding *T*. *solium* infections within rural Rwanda, particularly neurocysticercosis. Health education using the Vicious Worm education software should be considered in integrated control programs.

## Author summary

This research assessed the usefulness of a computer-based education tool for *Taenia solium* taeniasis/cysticercosis to train community health workers in Rwanda. The knowledge of health workers on the disease was assessed before the training, immediately after training, and four weeks later to determine baseline knowledge, knowledge uptake, and retention, respectively. The participants’ scores showed a significant improvement in knowledge immediately after the training. Four weeks later, the evaluation showed maintenance of the knowledge for at least the short term. Although digital literacy and lack of smartphones/computer to use the tool was noted as the main challenge, the participants highlighted the structure, completeness, and clarity of the information in the tool, recommending it be used in other areas.

## Introduction

Cysticercosis is a neglected tropical disease (NTD) caused by infection with the metacestode larval stage of *Taenia solium* and is associated with heavy public health and economic burden in marginalized areas of Africa, Asia, and South America [1,2]. Neurocysticercosis is a major cause of acquired seizures and epilepsy, and a recent study showed a high prevalence of cysticercosis in people with epilepsy in Rwanda [3].

Rwanda has a fast-growing pig production industry dominated by smallholder farmers who raise pigs for rapidly available income and to get manure for crop production [4]. Increases in smallholder pig production and pork consumption in sub-Saharan Africa are often associated with the emergence of porcine cysticercosis and, consequently, taeniasis and human cysticercosis [5]. Cysticercosis is known to be prevalent in pigs [6] and humans in Rwanda [3,7], and an analysis of pig value chains in Rwanda [4] showed sanitary practices embedded in the community to be inadequate, possibly due to various factors including lack of knowledge. The Rwanda strategic plan for NTDs highlights the lack of robust community awareness activities that would disseminate information on available preventive measures for cysticercosis [8]. Therefore, there is a need for additional methods targeting behavior changes through education.

Community health workers (CHWs) are considered by the World Health Organization (WHO) as important players for providing universal health coverage, especially to the most marginalized populations of low- and middle-income countries (LMIC) [9]. In Rwanda, the CHW started in 1995 after the Genocide against the Tutsi to respond to the unavailability of health infrastructures and enough health professionals. CHWs were elected by the community primarily during the monthly nationwide community service on the last Saturday of every month. CHWs must meet a few criteria, including the ability to read, calculate, and write, being from the community they are willing to serve, and serving as volunteers. In addition, they must not be a community leader or receive any remuneration from any health facility. They must be between 20 and 50 years old and have the community trust as an honest and reliable person [10]. In each Rwandan village (aggregation of about 50–200 households), three CHWs are designated. A pair of female and male CHWs known as agents de santé binôme which ensures integrated community case management of childhood illnesses, and a female CHW called Assistante Maternelle de Santé (ASM) taking care of maternal and newborn health services [10]. In Nyamagabe district, a 4^th^ CHW focuses on health promotion.

CHWs receive a three-month initial training; however, the training content, duration, and frequency are limited by available funds.

Since their deployment in 1995, CHWs have been at the center of important health-related achievements such as reduction of under-five and maternal mortality, improved family planning and vaccination coverage, as well as reduction of HIV/AIDS, malaria and tuberculosis [11,12]. Therefore, they could have an important role in tailored health education interventions for *T*. *solium* especially as education is increasingly being validated as an effective tool that complements other control and elimination programs [13,14].

The Vicious Worm educational tool (TVW) original English version, https://theviciousworm.be) was developed in 2014 by the University of Copenhagen [15]. TVW contains structured information on cysticercosis/taeniasis intended to support the training of a wide range of stakeholders ranging from school-age children to policymakers. Versions also exist in other languages spoken in Africa such as Swahili [16]. The novel digital-based tool is free and can be easily downloaded to smartphones or computers for teaching or self-study sessions. TVW has been shown to increase the knowledge of cysticercosis in pork supply chain workers and primary school children in Zambia [17–19]–but has not previously been tested in community health workers. The aims of the current study were to assess the baseline knowledge about cysticercosis in CHWs in an endemic area of Rwanda and then to evaluate the usefulness of the Kinyarwanda version of TVW for knowledge acquisition and retention.

## Materials and methods

### Ethics statement

This study was approved by the University of Global Health Equity Institutional Review Board (Reference: UGHE-IRB/2022/027). The participants provided their written informed consent to participate in this study. Before any data collection and training, the research objective was explained to CHWs who accepted our invitation. The research team explained that the participation was voluntary and emphasized that the participants could withdraw at any time without providing any justification. In the case of voice recordings, permission was obtained after explaining the reason for recording, which was to ensure that data collection was optimal, and that the participants’ thoughts were well captured.

### Study area

**Fig 1:**
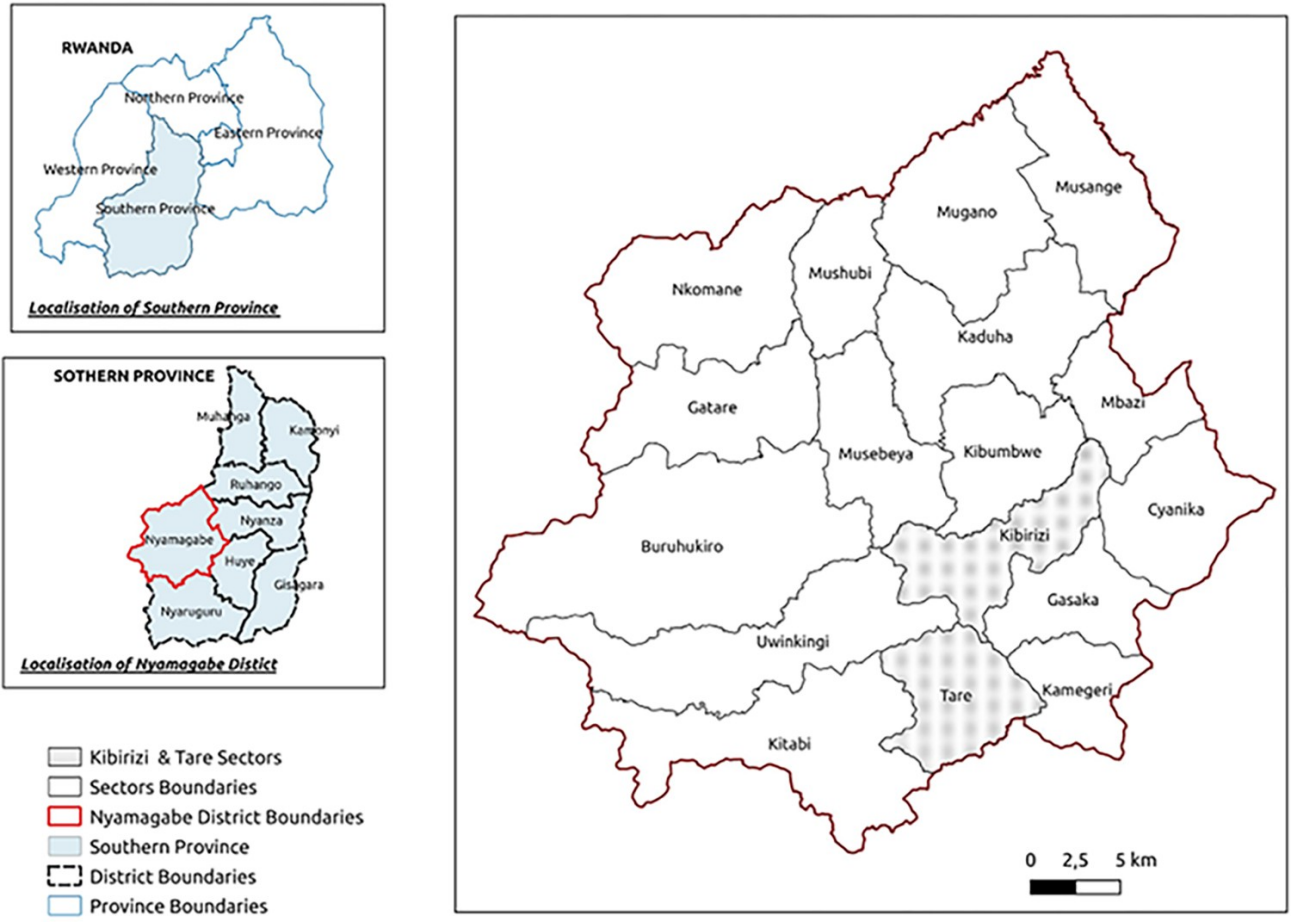
Map of Nyamagabe district and the two targeted sectors (QGIS Ver. 3.28—CC-BY license 4.0.). The layers are freely accessible from https://www.diva-gis.org/datadown and can be shared under CC-BY license 4.0.

Rwanda is a low-income country located in East Africa. It has a population of 13,246,394 and is one of the highest densities in Africa, with 501 inhabitants per sq. km [20]. Rwanda has a decentralized governance system designed to promote good governance, national development, and service delivery. Hence, the nation is geographically split into five provinces, including the City of Kigali. The provinces are subdivided into 30 districts. Furthermore, these districts are divided into 416 Sectors and further into 2,148 cells and 14,837 villages. This study was conducted in Nyamagabe, one of the seven districts of Southern Province, Rwanda (Fig 1). Nyamagabe has an area of 1090km^2^ with a total population estimated at 371,501, comprising 52.4% women and 47.6% men. The population is distributed in the district’s 17 sectors, 92 cells, and 536 villages, with an average density of 441/km^2^ [20].

Previous research highlighted sanitary risks that prompt the prevalence of cysticercosis and taeniasis in the area [4]. In addition, hospital records showed laboratory-confirmed cases of adult *tapeworm* infections in 236 and 334 patients in 2018 and 2019, respectively. This, coupled with the importance of pig production in the area, calls for interventions aiming at improving the knowledge on cysticercosis with the assumption that increased knowledge would trigger improvement in attitudes and practices and impact the disease endemicity in Nyamagabe.

### Study design

This longitudinal, cohort study design was carried out between May and July 2022. Data were collected using a structured questionnaire (S1 Table) and interview guide (S2 Table) initially designed in English. The questionnaire and the interview guide were translated into Kinyarwanda, and the answers were back translated into English. Before deployment to the study site, the questionnaire was pre-tested on volunteer CHWs selected in Kigali, and necessary adjustments were made.

The structured questionnaire was modified from the tool used by Ertel et al. [19], designed following the content of The Vicious Worm (TVW) education tool. It was composed of a total of 21 questions made up of four questions on the general knowledge of cysticercosis, seven questions that evaluated specific knowledge about porcine cysticercosis (PCC), six questions specific to taeniasis and finally, four specific questions about neurocysticercosis (NCC). The same questionnaire was used before the training using the TVW, immediately after, and four weeks later to evaluate pre-existing knowledge and knowledge uptake on cysticercosis/taeniasis.

### Selection of study participants, trainings, and data collection

Participants were recruited from two administrative sectors of Nyamagabe district, Kibirizi, and Tare. The sectors were randomly selected using the Excel randomization function, assuming a normal distribution of knowledge. The assumption was informed by our previous study on pigs and pork value chains [4]. All available CHWs were invited to participate, and the invitations were sent through the Nyamagabe Directorate of Health and CHWs reported at a meeting hall arranged by the district authority.

Before the training and data collection, TVW was translated into Kinyarwanda, the national language of Rwanda. The translations were carried out by the research team and the translations were checked for accuracy by two independent veterinarians, a public health expert and a medical doctor. The translated version can be accessed from https://theviciousworm.be.

The health education intervention was carried out using “The Vicious Worm” education tool. After evaluating the participants’ knowledge baseline, a three-hour health education session followed. All participants gathered in the same room and were given tablets with uploaded VWT. The research team used a computer and a projector to present the tool as they educated the participants about different sections of the tool. The tool’s content is organized into three parts: village, town, and city, and each part contains specific key messages dedicated to audiences found in the three settlements (village, town, and city). The participants were educated on the content of the village and town parts of the tool. The research team facilitated the participants’ navigation of the tool using the provided tablets and followed the content being explained and displayed on the projected screen. The participants were given time to ask questions during the presentations, and at the end of each part, they answered end-section revision questions in the tool.

The training took 3 hours, and at the end, according to the district’s guidelines, the participants were given food and drinks as well as a ticket refund for their travel expenses.

For qualitative data collection, we conducted two Focus Group Discussions (FGDs) with 8 participants for each FGD supplemented with in-depth interviews (IDIs) carried out with 10 participants that were not involved in the FGDs. The participants to the FGDs and IDIs were randomly selected from the cohort of CHWs who completed the follow-up questionnaire. FGDs and IDIs were conducted by the research team using semi-structured guides, and participants were asked to describe the following:

- If they were able to study the TVW content provided and how frequently per week.
- What they thought about TVW, its usefulness, and the perceived important sections covered by the software.
- What challenges were perceived while using TVW software.
- What improvements were needed to make the tool better for their counterpart CHWs.
- Any other thoughts they wanted to share about the tool.

### Data analysis and statistics

The answers to the questions included in the study were used to evaluate the participants’ knowledge before, immediately after, and four weeks after the training. Each correct answer was marked as 1 point, whereas a wrong answer was given a zero. For multiple-choice answers, a zero was given for any wrong answer selected, irrespective of the other answer(s) chosen. Therefore, the total number of points for each individual was calculated for "pre," "post," and "follow up" (i.e., before, immediately after, and four weeks after the training, respectively). To test for associations between the knowledge at each time point and potential explanatory variables, Mann-Whitney U (to compare the medians of two groups) or Kruskal-Wallis test (to compare the medians of more than two groups) were used.

To estimate knowledge uptake, the difference between the "post" and "pre" scores was calculated, whereas to evaluate knowledge retention after four weeks, the difference between the "follow-up" and "post" scores was computed. These data were tested using the Wilcoxon signed rank test using R version 4.1.1 (R Core Team, 2021).

For the qualitative data, the audio recordings were carefully transcribed verbatim within 24–48 hours, verified by re-playing and reading the transcripts, and any discrepancies between the audio recording and the transcripts were resolved. The recordings were complemented with information collected through IDIs. These transcripts were translated from Kinyarwanda to English. Data organization, coding and analysis was carried out using Dedoose Version 9.0.54 (Los Angeles, California) qualitative analysis software. Thematic analysis was used to explore the perceptions of CHWs towards TVW as well as recommendations regarding the improvement of the tool. Codes were generated iteratively based on input from the questions in the guides as well as from emerging themes. Themes were identified by reading the transcripts and identifying similarities and differences, repetitions, categorizations as well as interpretations of the data [21].

## Results

### Study Participants demographics

In total, the study enrolled 217 participants (73.3% of all the CHWs in the two sectors). All 217 participants took the pre-intervention test; however, 11 participants withdrew from subsequent tests for several reasons (urgent need to go back home after the training (n = 4), unavailable at follow-up test (n = 7)). Therefore, data from 206 participants were considered in the subsequent analysis and discussions.

The 206 participants were from two sectors, Kibirizi (60.2%) and Tare (39.8%) of Nyamagabe district (Table 1). The study participants were composed of females (66.5%) and males (33.5%), aged between 22 and 68 years (median = 44.5 years), with most of them (94.7%) married. Most of the study participants (70.9%) had attended primary school only, while 23.3% and 5.8% had attended secondary school, or professional training, respectively. Study participants had worked as CHWs between less than a year and 27 years (median = 10 years).

### Knowledge of taeniasis/cysticercosis

Originating from a *T*. *solium* endemic area, all participants had already heard about or seen taeniasis/cysticercosis. Of the total, 18.4% reported that either they or a family member had experienced Taeniasis. In addition, 78.8% had seen cysts of *T*. *solium* in a slaughtered pig carcass, and 38% did not recall any specific measures such as meat condemnation, taken on the infected carcass (Table 1).

**Table 1:** Demographic profile of Community Health Workers that participated in the three VWT testing phases (N = 206).

Variables		Frequency	Percentage
Sector	Kibirizi	124	60.2
	Tare	82	39.8
Gender	Female	137	66.5
	Male	69	33.5
Age	< 40	66	32.0
	40–50	81	39.3
	>50	59	28.6
Marital status	Single	3	1.5
	Married	195	94.7
	Widow/Widower	8	3.9
Education	Primary	146	70.9
	Secondary	48	23.3
	Professional	15	7.3
Experience	< 5 years	75	36.4
	5–10 years	31	15.0
	> 10 years	100	48.5
History of Taeniasis	Yes	38	18.4
in family	No	168	81.6
I have seen cysticercosis in a slaughtered pig carcass	Yes, often (once a week)	14	6.8
	Yes, regularly (< once a week)	30	14.6
	Yes, rarely (a few cases per year)	128	62.1
	No	24	11.7
	Does not know	3	1.5
	No answer	7	3.4
Decision on the seen positive carcass*	Nothing was done about it	83	38.2
	The infected part of the pig was removed	18	8.3
	The pig was treated with ash or salt	8	3.7
	The whole pig was destroyed	70	32.3
	Does not know	38	17.5

*Multiple answers allowed

The median score before the training was 15/21 (71.4%), with the participants individual scores ranging from 2/21 (9.5%) to 21/21 (100%). The detailed results of the study participants are shown in S3 Table.

The CHWs’ sector of origin was significantly associated with knowledge at baseline (*p* < 0.001), with median scores of 13.5/21 (64.3) in Tare and 15/21 (71.4) in Kibirizi. There was no other significant association across the participants’ demographic groups.

**Fig 2:**
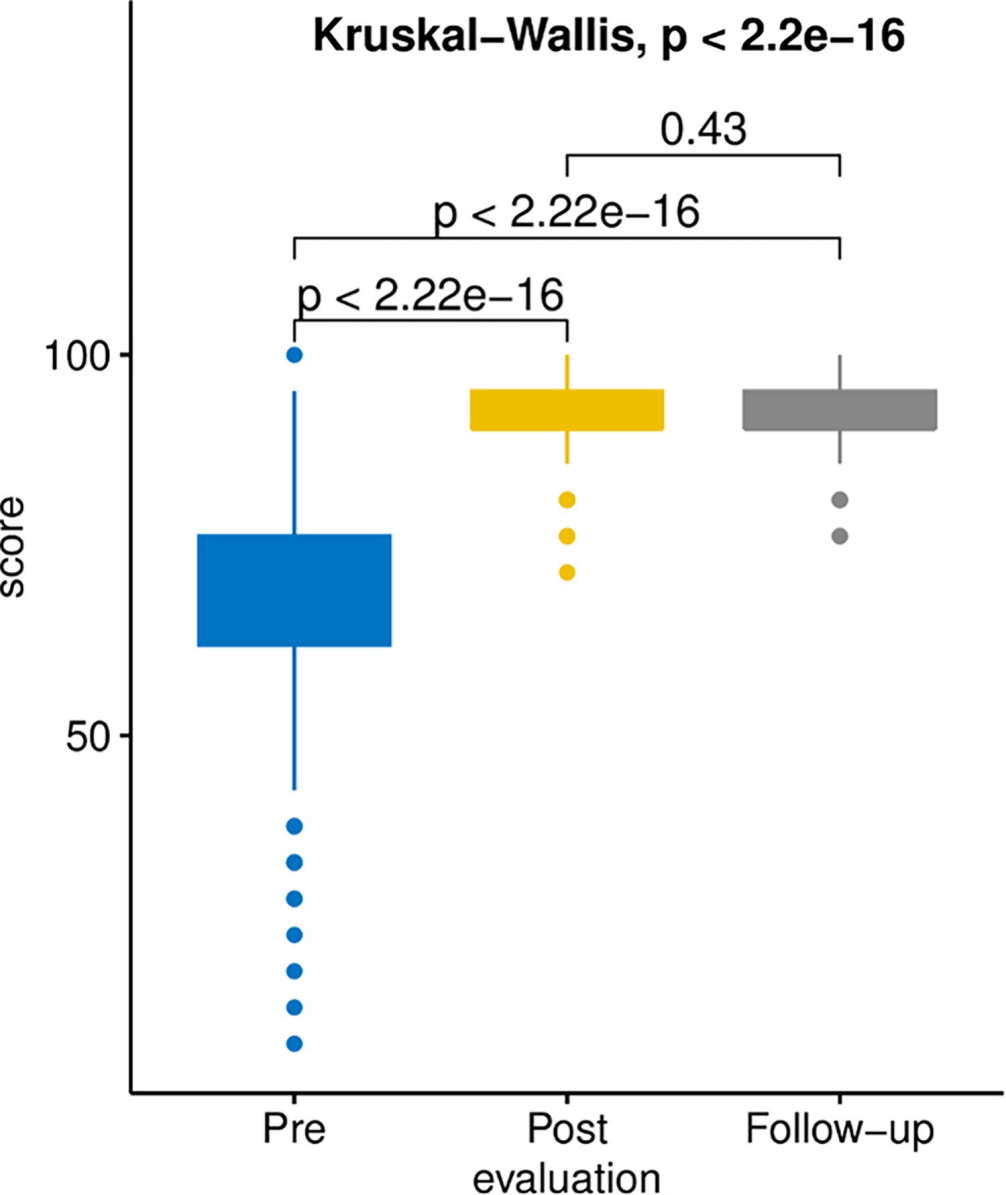
Percentage of overall score obtained for the three evaluations done.

The score improved significantly to a median score of 19/21 (90.5%) after the training (*p* < 0.001) and was maintained at the same level (90.5%) four weeks later (*p* < 0.001). The score ranged between 15/21 (71.4%) and 100% immediately after the intervention; and 16/21 (76.2%) and 100% at follow-up (Fig 2 and Table 2).

**Table 2:** Effects of the TVW on Knowledge Acquisition and Retention among Community Health Workers in Rwanda.

Category (n)	Pre_score (Range)	Post_score (Range)	Score at Follow up (Range)
General (n = 3)	2 (0–3)	3 (1–3)	3 (1–3)
Cysticercosis (n = 8)	6 (0–8)	8 (6–8)	8 (7–8)
Taeniasis (n = 6)	6 (0–6)	6 (4–6)	6 (4–6)
Neurocysticercosis (n = 4)	1 (0–4)	3 (1–4)	3 (1–4)
Total	15 (2–21)	19 (15–21)	19 (16–21)

When the score is broken down by themes evaluated, general aspects of cysticercosis scored 75.1% at baseline and increased to 83.8% and 82.7% at post and follow-up, respectively. For taeniasis-related questions, 87.2% of correct answers were recorded at baseline, and the score improved to 97.8% and 98.5%, respectively, for the post and follow-up sessions.

For aspects related to human cysticercosis and neurocysticercosis, 67.2% and 28.8% of correct answers were recorded at baseline, respectively. The knowledge related to the two aspects was increased after the training, and participants registered 97.1% and 77.9%, and the scores were sustained at 4-week follow-up with 99% and 72.6%, respectively (Table 2).

Participants had generally good knowledge on taeniasis, but much lower levels of knowledge on human cysticercosis, especially neurocysticercosis. For example, only 63/206 (30.6%) of participants could correctly answer whether humans can get cysticercosis. Correct answers increased with 200/206 (97.1%) at post-training assessment and 206/206 (100%) when the same question was asked at follow-up. Similarly, only 31/206 (15%) of the participating CHWs could accurately define neurocysticercosis. The proportion of good answers improved with 204 (99%) and 197/206 (95.6%) at post- and follow-up evaluation, respectively. Moreover, only 14/206 (6.8%) participants could tell how a person can get neurocysticercosis compared to 177/206 (85.9%) and 150/206 (72.8%) participants at post- and follow-up, respectively.

### CHW’s perceptions towards the usefulness of TVW

Our qualitative data analysis identified three recurring themes that guided the understanding of CHWs perceptions towards the education tool and needed adjustment for optimal use in field conditions. The three themes were:

- TVW is designed to promote education and comprehension of various aspects related to cysticercosis/taeniasis.
- TVW provides other valuable tips, such as proper pig husbandry, needed for socio-economic development and reduction of health risks and may speed up community change in attitude and practice toward cysticercosis.
- Some levels of digital literacy and access are key to the optimal use of TVW.

## 1. TVW promotes education and comprehension

The participants reported that TVW was informative, precise, and coherent. They found that the tool had various concepts about taeniasis, porcine cysticercosis, and neurocysticercosis that were new to them and yet explained in simple terms using their native language.

CHWs acknowledged that the most striking finding was that humans could also suffer from cysticercosis and neurocysticercosis.

*"The part of the tool that was most useful for me was where I learned the difference between porcine cysticercosis and human cysticercosis*. *This was the first time I learned that humans can also get cysticercosis*. *That part was useful to me*, *especially when teaching it to someone else in my village; they were very curious and happy to learn it*."

The tool provided knowledge and confidence to the participants, and they intended to use it against myths and misconceptions about cysticercosis. The tool is helpful as a guide to teaching others and ensures effective dissemination of knowledge within rural communities. It is easy to navigate with a good flow, has well-structured successive topics, and its visual contents and short stories encourage the reader to progress their reading and promote learning.

*"The training also helped me; for example*, *in my village*, *we learned that eating poorly cooked meat can infect you*. *We also learned that not using the toilets appropriately and allowing pigs access to human faeces exposes them to being infected with T*. *solium eggs*, *thus acquiring cysticercosis*. *In addition*, *we learned that eating vegetables we get from the markets without washing them well can infect us and get neurocysticercosis*".

*"I will use the tool to teach the community about cysticercosis preventive measures*. *In my village*, *when a CHW is given an opportunity to speak*, *they say they will share the educative materials they gained from the tool*. *We will teach the community members how to look for signs of cysticercosis in a pig and mobilize them to destroy the carcass if a pig is slaughtered and found to have cysticercosis*. *We will convey the message that infected pigs should not be eaten under any circumstances*".

*"People from my village get curious to read the tool when they see the images and short stories*. *For example*, *the story of someone who goes to buy a pig and asks the owner of the pig to first test for cysticercosis by checking the tongue*."

A CHW provided further explanations on the widespread lack of knowledge in people working along the pig value chain and stressed how the tool could help to address some gaps:

*"I once went to a pig slaughter slab to train*, *and they all said*, *"We are done; we didn’t know that cysticercosis is inside the meats; we thought it only exists on the tongue*. *We never thought it could also be in the muscles*.*" The tool taught us that cysticercosis can exist in any body part of the pig*."

## 2. TVW teaches proper pig husbandry for socio-economic development and the reduction of health risks

The CHWs reported that TVW prompted changes whether themselves, members in their households, or their communities after learning from the tool. The tool enlightened them about the dangers of cysticercosis as well as the risks associated with poor pig farming practices and poor hygiene practices on their health. They learned that inadequate practices are responsible for the health problems witnessed within their communities. This inspired them to be ambassadors of change in the practices within their communities.

To highlight the above point, one CHW reported:

*"I learned that the best way to build a pig pen is to put planks on the floor and to provide a covered roof so that pigs can find a shed away from the sun*. *I also learned how the pen should have a place for feeding and where to put water*. *Lastly*, *I also learned not to release pigs because that is how they get infected*."

The tool also provided knowledge on how best to ensure pig husbandry for a profitable business. A participant explained:

*"You see*, *now I know that if I want to start a project on pig farming*, *I will look for good breeds and try to find where I can get healthy pigs that are not infected by cysticercosis*. *I also know good farming practices like building a good pen for the pigs and not letting them roam freely because it could lead them to contracting cysticercosis*."

## 3. Digital literacy and access are key to the optimal use of TVW

The participants consistently raised digital illiteracy as a challenge they faced during the time they were attempting to use TVW. Most of them mentioned that they had no previous experience using a smart device, thus being stuck at every step as they tried to navigate through the tool. However, they mentioned that when they were facilitated, the navigation of the tool turned out to be easy, and they were able to lead their own revision of the materials independently. They also recommended the addition of more audiovisuals to each section of the tool to serve illiterate audiences. Furthermore, the participants expressed the need for regular workshops for continuous learning. Other recommendations included scaling up the implementation of the tool to educate the many communities in Rwanda and other countries.

To show the need for expanding the training to other actors and areas, participants explained:

*"It would be very important to other health care providers elsewhere in the country because even ourselves we did not know about this*. *Therefore*, *it would be important to them and their people*."

*"Yes*, *like in Cyangugu [south-west Rwanda]*, *there are a lot of pigs; I don’t know any other place with a lot of pigs*, *but the tool can also be used there and generate a positive impact*."

In the absence of smartphones, they suggested the provision of hard copies of the tool in the form of a booklet to facilitate them to revise the materials while they are in their homes. They reported that a well-printed booklet would help them to quickly disseminate the information since most of them do not own any kind of smart device through which they can access the tool regularly.

*"The way to improve this tool is that you would make it into a booklet so that my colleagues and I can easily access it*, *memorize the content on respective pages and use it comfortably*."

## Discussion

Health education can be an excellent weapon to support the elimination of *T*. *solium* [13,22] as a public health problem. CHWs are essential actors in healthcare systems, and they are the closest professional group to the population. They can act as agents of communities’ behavior change in Rwanda when they are effectively trained with the proper knowledge and given the right tools [23]. Community Health Workers are essential members of the community that can drive change through participation in awareness campaigns, early detection of disease symptoms in the community (on people and animals), as well as serving as a vessel of prevention and response information to the public health governing bodies. To be able to do so, the CHWs need to be trained and provided with adequate information that enables them to communicate effectively and without ambiguity, and to continuously improve their skills through continuing professional development.

The current study recruited CHWs in a cysticercosis-endemic region in the district of Nyamagabe in southern Rwanda. The overall baseline knowledge about several aspects of *T*. *solium* biology and public health risk was good, with higher scores associated with questions related to general knowledge, porcine cysticercosis, and taeniasis. However, participants scored poorly on questions about human cysticercosis and neurocysticercosis, highlighting the clear need for further training. Knowledge of porcine cysticercosis is generally good in the local population of endemic areas [24,25] as also shown here. This is not surprising because 83.5% had reportedly seen *T*. *solium* cysts in slaughtered pigs, while 18.4% of the participants had had Taeniasis (infection with the adult worm in the gut). However, quite a high proportion of participants were confused about the details of how pigs acquire infection: more than 40% (87/206) thought that a pig could acquire cysticercosis after mating with an infected pig, while 44% (90/206) believed that some pigs are born with the disease.

Despite the participants’ quite good knowledge of porcine cysticercosis and taeniasis and their experience with the parasite, specific knowledge about human cysticercosis and especially neurocysticercosis was lacking. This pattern has been observed by other studies carried out in India, Mexico, Kenya, and Tanzania [14,19,24,26–29]. The life cycle of *T*. *solium* is complex and can be difficult to understand and explain [15], especially as this parasite is associated with three related conditions: porcine cysticercosis, human taeniasis and human (neuro)cysticercosis, and with both environmental transmission and food-borne transmission elements. These details are often confusing for the public and may appear even more complex when new learners hear that human cysticercosis can evolve into neurocysticercosis and go on to cause symptoms which include epilepsy.

Knowledge of the disease was greatly improved in all those trained with TVW tool, at least over the time scale which we assessed, as has been found in other studies in Tanzania [19] and Zambia [17,18] where TVW tool was used in health education. At our follow-up, the acquired knowledge was maintained, however as this was only after 4 weeks, there is a need for longer term follow-up.

As a digital-based tool, TVW has the advantage of conveying standardized messages that can be adapted to different levels from the community to policymakers. As reported by our own participants, the tool has the advantage of being visual with easy-to-understand messages. Also, participants commended the inclusion of non-directly human health-related information, such as husbandry practices and economic aspects behind properly keeping pigs. Pig production is a mechanism of saving for many smallholders in Rwanda and other countries [4,30]. Thus, health education should include aspects that attract the interest of the community using real-life experience as part of the routine life of the trainees. In addition, using standardized message helps in avoiding unintended consequences such as associating epilepsy with pigs and thus deterring people from engaging in safe pig production.

The digital educational tools could, however, be challenging to operate, especially in rural areas where prior experience using smartphones is lacking. This was a consistent report from the study participants who seemed intimidated by the tool at the start of the training, although they got better at using it throughout the training. A previous study [23] that investigated opportunity costs and benefits among CHWs reported that some CHWs suggested that receiving smartphones would help them submit reports via the Internet, thus cutting transportation fees. In fact, if provided, smartphones can also be used for CHWs self-trainings and during their involvement in community awareness activities, using an educational tool like TVW. Phones are increasingly considered essential tools for a community-based surveillance system [31]. Hence, equipping community members such as CHWs could provide additional tools for community interventions, including awareness campaigns on health issues such as *T*. *solium*. There have been efforts aiming at enhancing CHWs access to smartphones to boost healthcare service [32,33]. With various initiatives that provide CHWs with phones, digitalization is expected to improve with time.

## Conclusion

This study confirmed a low level of proper understanding of *T*. *solium* transmission cycle, even in an endemic region. Using TVW, we demonstrated that knowledge can rapidly be improved and maintained in a short-term period. CHWs are essential actors in Rwanda’s health systems and are part of the key cadres of staff who could be involved in the elimination of NTDs in Rwanda. Therefore, equipping them with proper knowledge of the transmission and prevention of diseases is an essential aspect of the disease response mechanism. Including community animal health workers and community workers in charge of water and hygiene would also be important. Health education using TVW has proven effective in providing *T*. *solium* knowledge acquisition among CHWs and should be considered in integrated control programs.

## Supporting information

S1 Table Study Questionnaire Tool for the evaluation of knowledge about T. solium cysticercosis/Taeniasis. (DOCX)

S2 Table Interview Guide. (DOCX)

S3 Table Complete results from the assessment carried out with CHWs in southern Rwanda. Assessments were carried out before the training (“Pre”), immediately after the training (“Post”) and 4 weeks later (“Follow-up”).
(DOCX)
