# Supplementary material for: The Vicious Worm education tool improves the knowledge of community health workers on Taenia solium cysticercosis in Rwanda
Source: PLoS Negl Trop Dis. 2024 Apr 17;18(4):e0012140. doi: 10.1371/journal.pntd.0012140 (PMC11057718; doi:10.1371/journal.pntd.0012140)
Supplement: S1 Table — (DOCX) [file pntd.0012140.s001.docx]

**S1 Table.** Study Questionnaire Tool for the evaluation of knowledge about *T. solium* cysticercosis/Taeniasis.

| **Demographics** |
| --- |
| District |
| Sector |
| Cell |
| Village |
| Sex |
| Age |
| Marital status |
| Level of education |
| Function |
| How long have you worked as a CHW? |
| History of taeniasis and/or cysticercosis |
| Have you ever seen cysticercosis in a slaughtered pig carcass |
| If yes, what was the decision taken on the positive carcass |
| **General Knowledge** |
| Can people contract diseases from pigs? |
| If yes, what diseases do you know that people can contract from pigs? |
| Has any of your household members suffered from Taeniasis or Cysticercosis? |
| Do you think meat inspection should be performed? |
| **Cysticercosis** |
| Have you ever heard about Cysticercosis? |
| Can people get Cysticercosis? |
| How can a pig become infected with Cysticercosis? (Choose all options that apply) |
| a. From dirt getting into wounds |
| b. From eating human stool |
| c. From eating moldy maize bran or rice |
| d. From mating with an infected pig |
| e. Born with it |
| f. I don't know |
| How can you test for Cysticercosis in a live pig? |
| How can you test for Cysticercosis in a slaughtered pig? |
| What must be done with a slaughtered pig with porcine Cysticercosis? |
| What does Cysticercosis look like? |
| How can you prevent the pig from getting Cysticercosis? |
| Do you think meat inspection should be performed? |
| Have you ever seen porcine Cysticercosis in a slaughtered pig? |
| What was done with the pig with porcine Cysticercosis? (Choose all options that apply) |
| a. Nothing was done about it |
| b. The infected part of the pig was removed |
| c. The pig was treated with ash or salt |
| d. The whole pig was destroyed / Ingurube yose yaratawe |
| e. I don’t know / Ntabyo nzi |
| **Taeniasis** |
| Are Cysticercosis and human tapeworm related? |
| How do people get tapeworm infection? |
| What is a possible methods to diagnose pork tapeworm infections in people? (Choose all that apply) |
| a. It cannot be diagnosed |
| b. By looking at a stool sample using a microscope |
| c. By looking in the ear canal using a specialized tool |
| d. By checking the skin for infected wounds or worms under the skin |
| e. I don’t know |
| A person with a pork tapeworm will spread many tapeworms’ eggs through … |
| How can pork tapeworm be treated in people? |
| How can you prevent pork tapeworm in people? |
| **Neurocysticercosis** |
| What is human Neurocysticercosis? |
| A person with Neurocysticercosis/cysticercosis may have got the infection by… |
| What should a person who experiences seizures or severe chronic headaches do? |
| Can a person with Neurocysticercosis transmit the disease to other people? |
