# Supplementary material for: The Vicious Worm education tool improves the knowledge of community health workers on Taenia solium cysticercosis in Rwanda
Source: PLoS Negl Trop Dis. 2024 Apr 17;18(4):e0012140. doi: 10.1371/journal.pntd.0012140 (PMC11057718; doi:10.1371/journal.pntd.0012140)
Supplement: S2 Table — (DOCX) [file pntd.0012140.s002.docx]

**S2 Table. Interview Guide**

**Questions**

1. I would like to learn more about you. Were you able to use “The Vicious Worm” education tool?

***Probes:*** *How did you find enough time to use TVW tool/Tell me more why you were not able to use it. Why was that important to you to use it again?*

1. How frequently did you use the tool per week?

***Probes:*** *What caused you to access the tool this many/few times? How long did it take for you to go through the tool alone.*

1. Tell me what you think about “The Vicious Worm” education tool

***Probes:*** *Can you give me an example why you think that way? Can you tell me more about that?*

1. Share your experience of the usefulness of this tool, and tell me about what you think are the most important sections of the tool

***Probes:*** *Tell me more why you selected those most important sections? Could you give example emphasizing the importance of the selected sections? Why are those sections important to you?*

1. Is the tool easy to navigate or user-friendly?

***Probes:*** *Could you explain more what motivated your response? Is your response consistent with the whole tool, or you think it could vary depending on the tool sections?*

1. What are the challenges that you faced while using the tool and how did you overcome them?

***Probes:*** *Could you elaborate more on why you thought so? Please give specific examples to support your answer. Why does that matter for users of TVW tool?*

1. How do you think the tool should be improved?

***Probes:*** *Please tell me more about your answer. Can you give me an example on what can be changed? Why do you think changing that part could improve TVW tool?*

1. Do you think that the tool could be useful to your fellow CHWs and CAHWs in other locations?

**Probes:** *Could you elaborate more on your answer? Why do you think CHWs, and others could find benefits using TVW tool? Could you give any example to support your thoughts?*

1. What else would you like to share with us regarding “The Vicious Worm” education tool?

**Probes:** *Could you give more examples to elaborate on your answer? Why do you think that matter for potential users of TVW tool?*
