# Supplementary material for: The Vicious Worm education tool improves the knowledge of community health workers on Taenia solium cysticercosis in Rwanda
Source: PLoS Negl Trop Dis. 2024 Apr 17;18(4):e0012140. doi: 10.1371/journal.pntd.0012140 (PMC11057718; doi:10.1371/journal.pntd.0012140)
Supplement: S3 Table — Assessments were carried out before the training (“Pre”), immediately after the training (“Post”) and 4 weeks later (“Follow-up”). (DOCX) [file pntd.0012140.s003.docx]

**S3 table.** Complete results from the assessment carried out with CHWs in southern Rwanda. Assessments were carried out before the training (“Pre”), immediately after the training (“Post”) and 4 weeks later (“Follow-up”)

|  | **Correct answer** | | | **Knowledge change** | | |
| --- | --- | --- | --- | --- | --- | --- |
|  | **Pre (%)** | **Post (%)** | **Follow-up (%)** | **Post vs Pre** | **Follow-up vs Pre-** | **Follow-up vs Post** |
| **General** | **75.1** | **83.8** | **82.7** | **8.7** | **7.6** | **-1.1** |
| Can people contract diseases from pigs? | 97.6 | 99.5 | 100 | 1.9 | 2.4 | 0.5 |
| What diseases do you know that people can contract from pigs? | 48.1 | 60.7 | 56.3 | 12.6 | 8.2 | -4.4 |
| Do you think meat inspection should be performed? | 79.6 | 91.3 | 91.7 | 11.7 | 12.1 | 0.4 |
| **Cysticercosis** | **67.2** | **97.1** | **99** | **29.9** | **31.8** | **1.9** |
| Have you ever heard about Cysticercosis? | 94.7 | 100 | 100 | 5.3 | 5.3 | **0** |
| Can people get Cysticercosis? | 30.6 | 97.1 | 100 | 66.5 | 69.4 | 2.9 |
| How can a pig become infected with Cysticercosis? (Choose all that apply) | 29.1 | 88.3 | 94.7 | 59.2 | 65.6 | 6.4 |
| How can you test for Cysticercosis in a live pig? | 92.2 | 100 | 100 | 7.8 | 7.8 | 0 |
| How can you test for Cysticercosis in a slaughtered pig? | 78.6 | 96.1 | 100 | 17.5 | 21.4 | 3.9 |
| What must be done with a slaughtered pig with porcine Cysticercosis | 79.6 | 99 | 99.5 | 19.4 | 19.9 | 0.5 |
| What does Cysticercosis look like? | 77.2 | 97.6 | 99 | 20.4 | 21.8 | 1.4 |
| How can you prevent the pig from getting Cysticercosis? | 55.8 | 98.5 | 99 | 42.7 | 43.2 | 0.5 |
| **Taeniasis** | **87.2** | **97.8** | **98.5** | **10.6** | **11.2** | **0.6** |
| Are Cysticercosis and human tapeworm related? | 82 | 95.1 | 100 | 13.1 | 18 | 4.9 |
| How do people get tapeworm infection? | 87.4 | 95.6 | 96.1 | 8.2 | 8.7 | 0.5 |
| What is a possible methods for the diagnosis of the pork tapeworm infections in people? (Choose all that apply) | 91.3 | 99 | 99.5 | 7.7 | 8.2 | 0.5 |
| A person with a pork tapeworm will spread many tapeworms’ eggs through … | 92.7 | 99.5 | 100 | 6.8 | 7.3 | 0.5 |
| How can pork tapeworm be treated in people? | 91.3 | 99.5 | 99 | 8.2 | 7.7 | -0.5 |
| How can you prevent pork tapeworm in people? | 78.6 | 98.1 | 96.1 | 19.5 | 17.5 | -2 |
| **Neurocysticercosis** | **28.8** | **77.9** | **72.6** | **49.2** | **43.8** | **-5.3** |
| What is human Neurocysticercosis? | 15 | 99 | 95.6 | 84 | 80.6 | -3.4 |
| A person with Neurocysticercosis/cysticercosis may have got the infection by… | 6.8 | 85.9 | 72.8 | 79.1 | 66 | -13.1 |
| What should a person who experiences seizures or severe chronic headaches do? | 80.6 | 99.5 | 100 | 18.9 | 19.4 | 0.5 |
| Can a person with Neurocysticercosis transmit the disease to other people? | 12.6 | 27.2 | 21.8 | 14.6 | 9.2 | -5.4 |
